# Reliability of a German version of the Kansas City Cardiomyopathy Questionnaire (KCCQ) administered via telephone

**DOI:** 10.1038/s41598-025-14179-6

**Published:** 2025-08-04

**Authors:** Martha Schutzmeier, Viktoria Rücker, Jonas Widmann, Anna Grau, Caroline Morbach, John A. Spertus, Jürgen Deckert, Stefan Störk, Peter U. Heuschmann

**Affiliations:** 1 Institute for Clinical Epidemiology and Biometry, Julius-Maximilian University Würzburg, Würzburg, Germany; 2 Department Clinical Research and Epidemiology, Comprehensive Heart Failure Center Würzburg, University Hospital Würzburg, Würzburg, Germany; 3 Department Medicine I, University Hospital Würzburg, Würzburg, Germany; 4 Kansas City’s Healthcare Institute for Innovations in Quality and Saint Luke’s Mid America Heart Institute, University of Missouri, Kansas City, MO USA; 5 Clinical Trial Centre Würzburg, University Hospital Würzburg, Würzburg, Germany; 6 Institute for medical Data Sciences, University Hospital Würzburg, Würzburg, Germany

**Keywords:** KCCQ, Heart failure, Quality of life, Reliability, Cardiology, Health care, Medical research

## Abstract

To date, there is no validated telephone version of the Kansas City Cardiomyopathy Questionnaire (KCCQ) available to collect data about health-related quality of life among patients with heart failure (HF). We assessed the reliability of the German KCCQ administered via telephone in comparison to the self-administered version. Patients with HF admitted to the outpatient clinic of the University Hospital Würzburg were consecutively identified and recruited. Patients completed (a) the self-administered version of the KCCQ, and (b) the telephone-based interview performed by trained raters. The sequence of both approaches was randomized. For the between-method agreement, the intraclass correlation coefficient (ICC) was calculated using a non-parametric, rank-based approach. We analysed data from sixty-one HF patients. The median KCCQ overall summary score was 84.8 (interquartile range (IQR) 71.6–76.9). The test-retest reliability between the self-administered and the telephone interview showed good agreement for the total symptom score, the clinical score and the overall summary core: ICC 0.75, 95 % confidence interval (CI) 0.72–0.79; ICC 0.80, CI 0.77–0.84; ICC 0.83, CI 0.80–0.86, respectively. The German KCCQ administered via telephone showed good test-retest reliability, indicating its applicability to collect data about health status among HF patients over the phone.

**Supplementary Information:**

The online version contains supplementary material available at 10.1038/s41598-025-14179-6.

## Introduction

Although the treatment and management of patients with heart failure (HF) has improved over the last 2 decades, its prognosis remains poor and the quality of life (QoL) of patients with HF remains limited, with frequent hospitalizations and compromised physical capacity^1,2^.

Quantifying patients’ disease-specific health-related quality of life (HRQoL) in HF is a guideline-recommended component of evaluating treatment success and ongoing care^3,4^. Patient-reported outcomes measures (PROMs) allow for systematic collection of health status and can be an important tool to improve patient-centered HF care^5^. Among patients with HF, the Kansas City Cardiomyopathy Questionnaire (KCCQ) has emerged as a valid means of quantifying the impact of HF from patients’ perspectives. The KCCQ is a self-administered, 12- or 23-item questionnaire that quantifies multiple health domains and can generate three summary scores^3^. The KCCQ-23 has been previously validated for the German language^6^. It is increasingly being used as an outcome in clinical trials, particularly after being qualified as a Clinical Outcome Assessment by the U.S. Food and Drug Administration (FDA) to support regulatory claims^7^.

Telemonitoring and tele-health consultations are widely used patient care strategies for patients with HF^8^. Telephone-based approaches are ubiquitously available strategies to assess the clinical status and alter treatment in patients with HF^9^. Augmenting these often unstructured clinical assessments with validated PROMs tools, such as the KCCQ, could potentially improve their validity and consistency. Even health status assessed by physicians using the New York Heart Association (NYHA) functional class is less accurate and prognostic than the KCCQ^10,11^. The KCCQ serves as a valuable tool for evaluating HF patients’ response to therapy and clinical risk, offering critical insights into health status that can help identify high-risk patients for timely referral and treatment modifications^12^. Additionally, establishing the validity of a telephone version of the KCCQ could support its use as an outcome in clinical trials where in-person visits are not feasible and mailed questionnaires are not returned. Data collection over the phone allows for iterative assessments and efficient use of economic and human resources, and could potentially impact positively in the development of relationships between researchers and participants, without compromising the quality of collected data^9,13^.

To date, there are no published data regarding the reliability of collecting the KCCQ over the phone. Thus, the aim of the present study was to develop a telephone version of the KCCQ-23 and to evaluate its reliability in comparison to a self-administered version.

## Methods

### Patient recruitment and data collection

Patients with heart failure from the outpatient clinics of the University Hospital Würzburg were consecutively identified and recruited. Inclusion criteria were age of at least 18 years, written informed consent, and a confirmed diagnosis of chronic HF with left ventricular ejection fraction (LVEF) ≤ 40% determined by echocardiography at the time of diagnosis. Patients were excluded from the study if they had advanced cognitive impairment (documented in the patients’ medical records or according to physicians’ impressions) or did not have telephone access (landline or mobile phone). Additionally, patients who were not sufficiently proficient in spoken and written German to comprehend the study materials and instructions were not eligible to participate in the study.

**Fig. 1:**
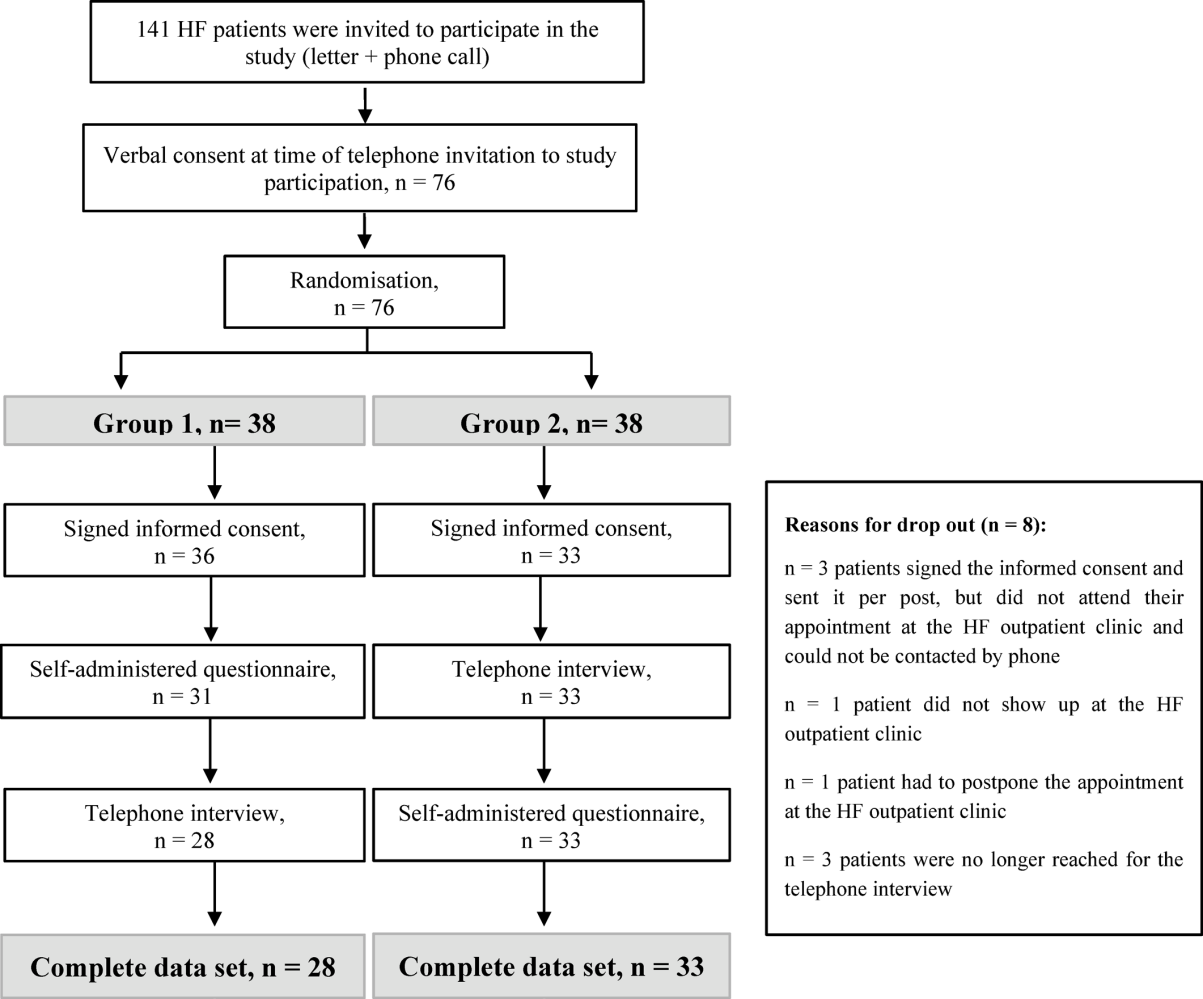
Recruitment and randomisation process of the patients.

Recruitment and data collection took place between July and October 2021. Figure 1 describes the recruitment and randomisation process of the patients. Patients were sent a letter approximately six weeks prior to their scheduled outpatient clinic appointment, which included the study invitation, patient information, two copies of the informed consent form, and a pre-addressed return envelope. Following this, patients were contacted by phone to confirm their interest in participation. Those who agreed were randomised and, depending on group assignment, patients were asked to either return the signed consent form by mail or bring it to their clinic appointment. One hundred and forty one patients were invited to participate in the study, 65 were not reached for the telephone invitation and 76 verbally agreed and were randomized to Group 1 (self-assessment questionnaire first) or in Group 2 (telephone interview first). Of the randomized patients, 69 signed the informed consent.

To test the reliability of the KCCQ administered via telephone, the questionnaire was completed once in person with each patient and once by phone. Thus, all patients completed the written self-reported KCCQ questionnaire in the outpatient clinic and were contacted by phone to complete the KCCQ over the phone with a project staff member. The sequence of the type of interviews (telephone or written) was randomized. The study protocol had pre-specified that the time interval between both administrations should be five days, as the recollection time for the KCCQ refers to a time interval of 14 days^3^. Therefore, after approximately 5 days, the second survey was administered in the form that had not yet been administered to the patient, in order to compare both forms of administration.

### Kansas City cardiomyopathy questionnaire 23 (KCCQ-23)

The KCCQ-23 quantifies seven domains of the patient’s HF related health status: Physical Limitation (6 items), Symptom Stability (1 item), Symptom Frequency (4 items), Symptom Burden (3 items), Self-Efficacy (2 items), Quality of Life (3 items) and Social Limitations (4 items).

The KCCQ-23 also quantifies three scores, as a result from the combination of the domains: Total Symptom score (average of Symptom Frequency and Symptom Burden), Clinical Summary score (average of Physical Limitation and Total Symptoms) and Overall Summary score (average of Physical Limitation, Total Symptoms, Quality of Life, and Social Limitation).

Each domain is scored from 0 to 100. Higher scores indicate fewer symptoms, less social or physical limitations and better quality of life, meaning a better health status^3^. A clinically important difference is indicated by a 5-point difference in the KCCQ-23 overall summary score between groups, as well as within individual patients^3,12,14^.

A validated shorter version of the KCCQ (KCCQ-12) is also available. The KCCQ-12 assesses only four domains - Physical Limitation, Symptom Frequency, Quality of Life and Social Limitation - and, like the KCCQ-23, is scored on a scale from 0 to 100^15^.

### Changes in the questionnaire for the telephone interview

The development of the telephone interview of the KCCQ was based on the German, self-administered, 23-item KCCQ^6^. The items, as well as their number, remained unchanged. The following minor changes or additions were made:

1. In the original version of the KCCQ-23, there is already a short description of the questionnaire at the beginning and instructions for the patient on how to fill in the questionnaire. For the telephone version, the duration of the interview and a brief explanation of the structure of the questionnaire were added to the description. The study participants were also informed that they should ask for a repetition of a question or answer if they did not understand it.
2. For questions 1 and 15, where several activities are mentioned for the same answer options, a short description of the question structure was added.
3. Regarding the remaining questions, a brief reference of the next topic was added. This change was made to simplify the transition from one question to the next for the study participants.

### Data analysis

Descriptive statistics were calculated for sociodemographic and clinical variables. Sociodemographic and clinical characteristics were compared between groups using Student’s t-test for continuous variables and Chi-square test for categorical variables as appropriate. KCCQ-23 scores were compared by form of administration using the Wilcoxon rank sum test. This test was also used to compare the between-group difference between the overall summary score of the first and second data collection. A Kruskal–Wallis rank sum test was used to compare the differences in overall summary scores between the first and second administrations across different time intervals. For all scores and domains, as well as in a subgroup analyses, agreement between the telephone- and the paper-based version was assessed by calculating intraclass correlation coefficients (ICC) with 95% confidence intervals (CI). A non-parametric, rank-based approach^16,17^ was calculated for the ICC, as the data was not normally distributed. Moreover, a sensitivity analysis was conducted, the KCCQ-12 scores were compared according to the form of administration using the Wilcoxon rank sum test, and the ICC was also calculated for the KCCQ-12. Using PASS 2020 a sample size calculation conducted prior to recruitment estimated that a sample size of 60 participants, each measured twice, would be needed to estimate an assumed good ICC of 0.7 with an acceptable precision of 12.8% (half-width of a 95%-CI). The analyses were performed using R version 4.2.2 (R core team, Vienna, Austria, 2022), except for the non-parametric ICC, which was calculated with MS Excel version 2016.

### Ethical considerations

Informed consent was obtained from all participants of the study. The study was performed in accordance with the Declaration of Helsinki and all methods were fully approved by the ethics committee of the Medical Faculty of the University Würzburg (No.: 27/21-am). The responsible data protection officer approved the data management concept.

## Results

The mean age of study participants was 60.4 ± 14.6 years, 71% were men, and 37% were in NYHA functional class II. Table 1 describes the basic demographic and clinical characteristics of the participants.

**Table 1:** Characteristics of the participants.

	Group 1 (self-administered questionnaire first)	Group 2 (telephone interview first)	*p*-value
Sex (*n* = 69)			
Men (n, %)	28 (77.7)	21 (63.6)	0.19
Age (*n* = 69)			
Mean (SD)	57.61 (16.2)	63.33 (12.2)	0.10
Left ventricular ejection fraction (LVEF) (*n* = 69)			
Mean (SD)	34.78 (9.8)	38.21 (12.7)	0.21
NYHA class (*n* = 68)			
I (n, %)	9 (25.0)	10 (31.2)	0.84
II (n, %)	14 (38.9)	11 (34.3)	
III and IV (n, %)	13 (36.1)	11 (34.3)	
Comorbidities			
Diabetes (n, %) (*n* = 68)	17 (48.5)	9 (27.2)	0.07
Hypertension (n, %) (*n* = 69)	21 (58.3)	24 (72.7)	0.20
Hyperlipidemia (n, %) (*n* = 61)	20 (55.5)	21 (63.6)	0.49
Depression (n, %) (*n* = 39)	4 (22.2)	6 (28.5)	0.728
Other comorbidities (n, %) (*n* = 68)	31 (86.1)	29 (90.6)	0.71

*LVEF* left ventricular ejection fraction, *NYHA* New York Heart Association.

**Fig. 2:**
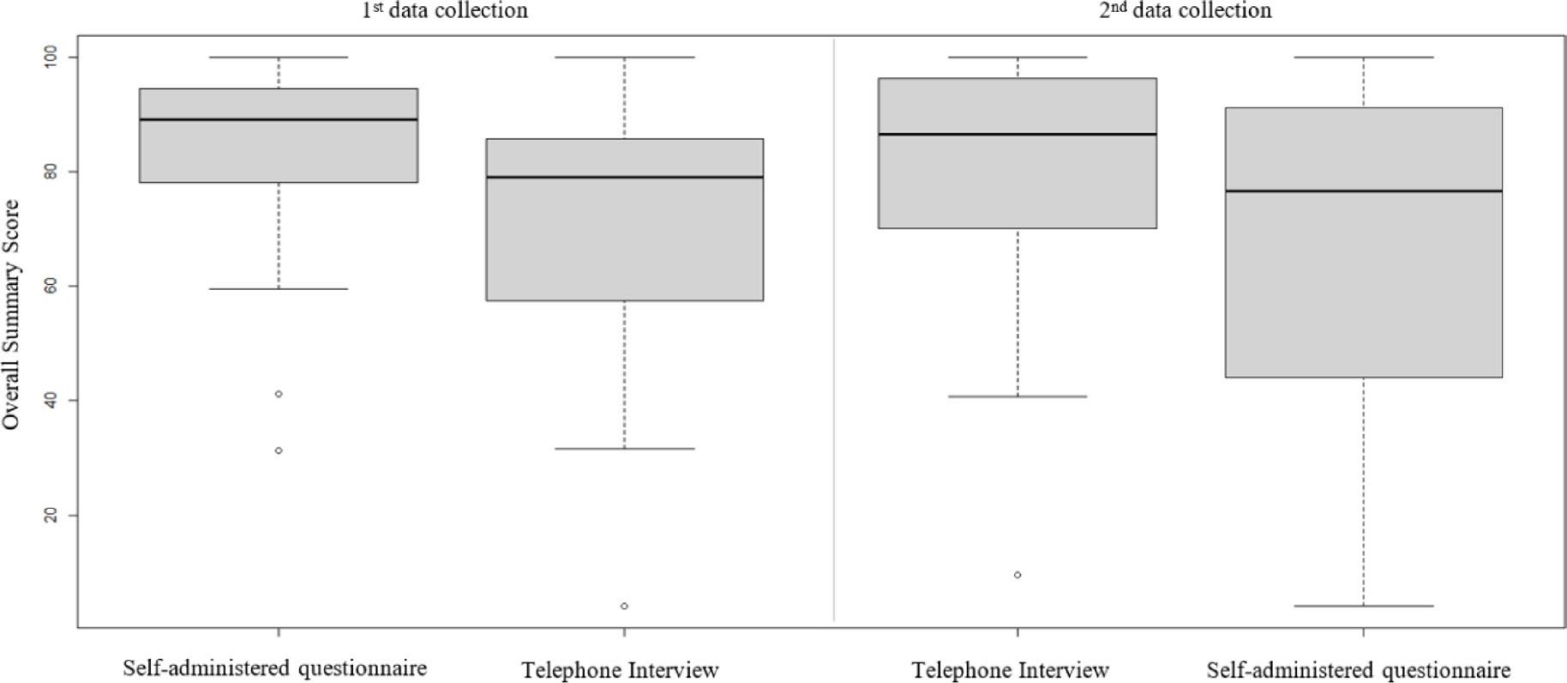
Results of the overall summary score for order and form of administration.

Only patients completing both questionnaires were considered for the data analysis regarding the KCCQ (*n* = 61). The interval between completion of both questionnaires was on average 5.7 ± 2.4 days and the telephone interview took on average 11.42 ± 6.8 min. Mean scores on the KCCQ scales ranged from 81 to 87, except for the domain Symptom Stability. Table 2 shows the score and domain values according to the form of administration. The KCCQ scores did not differ significantly between both groups, except for the domain Social Limitations. Figure 2 shows the results of the overall summary score according to order and form of administration. The difference of the overall summary score comparing first and second data collection between both groups was not statistically significant (*p* = 0.66). Supplemental Figs. 1 and 2 illustrate the relationship between the median of the overall summary score and the difference between forms of administration. In Supplemental Fig. 1, data points are color-coded by the order of administration, while in Supplemental Fig. 2, the different colors represent the time interval (in days) between the two assessments. The difference in overall summary scores across the different time intervals was not statistically significant (*p* = 0.05).

**Table 2:** Results of the KCCQ-23 for both ways of administration (*n* = 61).

	KCCQ (median, IQR)		
	Self-administered	Telephone interview	*p*-Value
Domains			
Physical limitation	83.33 (75–95.83)	79.17 (66.67–95.83)	0.90
Symptom stability	50 (50–50)	50 (50–50)	0.38
Symptom frequency	91.67 (66.66–95.83)	83.33 (62.5–100)	0.17
Symptom burden	83.33 (75–100)	83.33 (66.66–100)	0.08
Self-efficacy	87.5 (75–100)	87.5 (75–100)	0.12
Quality of life	83.33 (66.66–100)	83.33 (58.3–91.66)	1
Social limitations^a^	82.29 (56.25–100)	87.5 (68.75–100)	**0.005**
Scores			
Total symptom score	87.5 (72.91–97.91)	79.17 (63.54)	0.73
Clinical summary score	87.5 (70.13–94.44)	83.33 (66.66–93.05)	0.05
Overall summary score	83.96 (68.75–94.16)	84.17 (64.58–91.25)	0.71

*KCCQ* Kansas City Cardiomyopathy Questionnaire, *IQR* interquartile range.

^a^*n* = 60.

The test-retest reliability results showed general agreement between both ways of administration of the KCCQ. The ICC values showed good agreement for the Total Symptom Score (ICC 0.75, CI 0.72–0.79), the Clinical Summary Score (ICC 0.80, CI 0.77–0.84) and the Overall Summary Score (ICC 0.83, CI 0.80–0.86). The ICC for the domains of the KCCQ varied between 0.44 for the domain Symptom Stability and 0.84 for the domain Quality of Life. Table 3 describes the results of the rank-based approach calculation and Table 4 the results of the rank-based approach calculation for each group. The test-retest reliability for both groups also demonstrated good reliability for all three scores of the KCCQ, except for the Total Symptom Score in group 1, which showed moderate reliability (ICC 0.60, CI 0.52–0.67).

**Table 3:** Test-retest reliability between telephone-assessed and self-administered KCCQ-23 (*n* = 61).

	ICC	95%-CI
Domains		
Physical limitation	0.65	0.62–0.69
Symptom stability	0.44	0.41–0.47
Symptom frequency	0.77	0.74–0.80
Symptom burden	0.69	0.66–0.72
Self-efficacy	0.66	0.63–0.70
Quality of life	0.84	0.81–0.88
Social limitations^a^	0.67	0.64–0.71
Scores		
Total symptom score	0.75	0.72–0.79
Clinical summary score	0.80	0.77–0.84
Overall summary score	0.83	0.80–0.86

*KCCQ* Kansas City Cardiomyopathy Questionnaire, *ICC* non parametric intraclass correlation coefficient, *CI* confidence interval.

^a^*n* = 60.

**Table 4:** Test-retest reliability between telephone-assessed and self-administered KCCQ-23 for both groups (*n* = 61).

	Group 1 (*n* = 28) (self-administered questionnaire first)		Group 2 (*n* = 33) (telephone interview first)	
	ICC	95%-CI	ICC	95%-CI
Domains				
Physical limitation	0.82	0.75–0.90	0.46	0.40–0.52
Symptom stability	0.57	0.50–0.65	0.28	0.22–0.34
Symptom frequency	0.66	0.58–0.73	0.82	0.75–0.88
Symptom burden	0.56	0.478–0.63	0.74	0.68–0.81
Self-efficacy	0.87	0.79–0.94	0.49	0.43–0.55
Quality of life	0.85	0.78–0.93	0.80	0.74–0.87
Social limitations^a^	0.62	0.54–0.70	0.66	0.59–0.72
Scores				
Total symptom score	0.60	0.52–0.67	0.81	0.74–0.87
Clinical summary score	0.81	0.74–0.89	0.78	0.72–0.84
Overall summary score	0.84	0.76–0.91	0.82	0.76–0.88

*KCCQ* Kansas City Cardiomyopathy Questionnaire, *ICC* non parametric intraclass correlation coefficient, *CI* confidence interval.

^a^Group 1 (*n* = 27).

The sensitivity analysis demonstrated that there was no statistically significant difference between the domains and scores of the KCCQ-12, according to the form of administration, as shown in Supplementary Table S1. Furthermore, the ICC values for the KCCQ-12 scores ranged from 0.67 to 0.85 for the Physical Limitation Score and the Overall Summary Score, as presented in Supplementary Table S2.

## Discussion

To improve the efficiency of care and feasibility of directly quantifying patients’ perceptions of their health status, defining better methods for collecting patient-reported outcomes is needed. In HF, the KCCQ is a widely used patient-reported outcome measure that is increasingly being used in clinical research and clinical care. Our study showed that the KCCQ administered via telephone had good test-retest reliability, with ICCs for all three summary scores being high and thus supporting the validity of data collected by phone, as compared with self-administration.

Faller et al.^6^ validated the German version of the KCCQ and reported high internal consistency (Cronbach’s alpha = 0.56–0.93) and excellent test-retest reliability (ICC = 0.79–0.94) across its domains and scores. Construct validity was supported by strong correlations with corresponding subscales of the Short Form-36 Health Survey (SF-36), a validated instrument for assessing health status. To assess test-retest reliability, a clinically stable subsample was identified, and the KCCQ was administered twice over a one-week interval. These results align with the findings of our study, which also demonstrated good test-retest reliability, with ICC values ranging from 0.44 to 0.84, when comparing both forms of administration.

There are a number of advantages to collecting data on HRQoL by phone, such as the possibility of more frequent assessments and efficient use of resources^9,13^. Yet, up to now, published data on the reliability of collecting HRQoL data over the phone is lacking. McPhail and colleagues investigated the agreement between telephone and face-to-face administration of two instruments, one assessing functional independence (Frenchay Activities Index (FAI)) and one assessing HRQoL (Euroqol-5D (EQ-5D)), amongst patients aged ≥ 65 years. Their findings indicated high levels of agreement at the individual item level and overall scores for each method of administration. These authors found that using the telephone to collect information in this population may be a valid and a more efficient and convenient alternative to traditional in-person assessments. Especially for older adults who have adequate basic cognitive functioning, and may provide a more accessible and less time-consuming means of collecting data^18^.

Other studies have also shown that a telephone interview is comparable to the in-person application of different HRQoL questionnaires. Rocha et al. evaluated the reliability of three different questionnaires administered by telephone interview in a population of patients with chronic obstructive pulmonary disease (COPD). The authors found that the phone-based data collection is valid and reliable, and therefore can be an alternative approach to in person interviews for monitoring symptoms and HRQoL in patients with COPD^19^. Another study from Goetti et al. developed a numerical scale (NS) version of the Western Ontario Shoulder Instability Index (WOSI), a 21-item questionnaire designed to assess the QoL in patients with shoulder instability. The objective of the study was to evaluate the reliability and consistency of administering the questionnaire via telephone and email. The findings demonstrated that there were no statistically significant differences in the performance of the NS-WOSI, irrespective of whether it was administered in person or via telephone. However, telephone-based data collection was found to be more reliable, as evidenced by higher ICCs, and time-efficient than email administration, leading the authors to recommend telephone over email administration^20^. In our study, there was no statistically significant difference in the overall summary score comparing first and second data collections between both groups. We also found a good agreement for all scores of the KCCQ between telephone interview and self-assessment. The ICC for the different domains Physical Limitation, Symptom Frequency, Symptom Burden, Self-efficacy, Quality of Life and Social Limitations demonstrated a moderate to good reliability, varying from 0.65 to 0.84. The reliability of the Symptom Stability domain was lower, possibly because it is measured with only one item. However, this domain was not included in the calculation of the Overall Summary Score, or of the other scores of the KCCQ^3,12,14^.

Another study by Chatterji et al. found equivalence between the telephone and self-completed modes of administration for the EuroQol survey. However, their study revealed that there was inconsistent agreement among the scores of individual domains. Therefore, the authors did not recommend interpreting changes in individual questions over time if different data collection methods were applied to administer the EuroQol survey^21^. Our results yielded a similar issue with the domains of the KCCQ, particularly in the subgroup analysis. The agreement between domains for the self-administered questionnaire and the telephone interview varied to a larger degree than the scores. When multiple data collection methods have been employed, it is essential to take this into account when interpreting the data. Nevertheless, the utilization of two distinct data collection methods is still preferable to the potential issues associated with missing data. Furthermore, the subgroup analysis also showed a good agreement between the form of administration for all scores of the KCCQ in both groups, with the exception of the Total Symptom Score in group 1, that showed moderate agreement.

Our results showed a statistically significant difference in the Social Limitations domain of the KCCQ between self-assessment and telephone interview. This domain includes questions about personal topics such as intimate relationships, hobbies, household and workplace responsibilities, and visits to family and friends outside their own home. These questions may be subject to social desirability bias^22^which might have played a role during the telephone interview. An alternative to this issue could be the use of internet-based administration of the KCCQ. Wu et al. demonstrated in their study that internet administration is equivalent to self-administration for the KCCQ^23^.

In Germany, since January 2022, remote patient monitoring (RPM) for HF patients is covered by statutory health insurance (SHI)^24^. To enter the RPM care pathway and get RPM reimbursed by the SHI, patients must meet specific criteria to, e.g. NYHA functional class II or III and treatment according to guidelines. A regular assessment of health status using the KCCQ could be included in routine follow up calls as part of telemonitoring in HF. In combination with information from RPM, it could improve the care of HF patients. This is especially important when patient care is provided by more than one provider, as they may assign different NYHA functional classes to the same patient^12^. The assessment of health status by referring to the KCCQ scores is a more accurate method of detecting meaningful changes in health status over time than changes in NYHA functional class, thereby offering greater prognostic value^11^. Furthermore, physicians have a standardized reference to assess the patient’s condition^12^.

### Strengths and limitations

To the best of our knowledge, this study was the first to evaluate the reliability of the KCCQ assessment over the phone. The results indicated good agreement for all three KCCQ scores between the telephone interview and the self-assessment questionnaire. The time between interviews should be short enough to avoid changes in health status, but long enough to exclude any memory effects. In our study, the time between administrations of both versions of the KCCQ was on average 5.7 ± 2.4 days, ensuring there were likely no changes in the health status of the participants between assessments.

Our study has limitations, as it is monocentric and has a relatively small number of cases. However, the sample size was adequate to answer the research question, as estimated by the sample size calculation. A further limitation of the study is the restriction of the study sample, i.e. the exclusion of patients who were unable to be interviewed by telephone. However, this was due to the objective of the study and therefore unavoidable. Another limitation of our study is that the telephone interview was conducted by a member of the research team, which may have introduced social desirability or interviewer bias.

## Conclusion

Our findings provide results supporting the collection of the KCCQ over the phone, which can facilitate monitoring HF-specific health status in routine care and in large-scale clinical research. We believe that KCCQ data collection over the phone could also work in other languages and countries, but future research should confirm this expectation.

## Supplementary Information

Below is the link to the electronic supplementary material.

Supplementary Material 1

## Data Availability

The datasets used and/or analysed during the current study are available from the corresponding author on reasonable request.
