# Supplementary material for: Reliability of a German version of the Kansas City Cardiomyopathy Questionnaire (KCCQ) administered via telephone
Source: Sci Rep. 2025 Aug 4;15:28444. doi: 10.1038/s41598-025-14179-6 (PMC12322219; doi:10.1038/s41598-025-14179-6)
Supplement: Supplementary file 1 — Supplementary Material 1 [file 41598_2025_14179_MOESM1_ESM.docx]

**Supplementary Information**

**Article title:** Reliability of a German telephone version of the Kansas City Cardiomyopathy Questionnaire (KCCQ)

**Journal name:** Scientific Reports

**Author names:** Martha Schutzmeier^1*^, Viktoria Rücker^1^, Jonas Widmann^1^, Anna Grau^1^, Caroline Morbach^2,3^, John A. Spertus^4^, Jürgen Deckert^1^, Stefan Störk^2,3^, Peter U. Heuschmann^1,5,6^

**Affiliations:**

^1^ Julius-Maximilian University Würzburg, Institute for Clinical Epidemiology and Biometry, Würzburg, Germany

^2^ Department Clinical Research & Epidemiology, Comprehensive Heart Failure Center Würz-burg, University Hospital Würzburg, Germany

^3^ Department Medicine I, University Hospital Würzburg, Germany

^4^ University of Missouri – Kansas City’s Healthcare Institute for Innovations in Quality and Saint Luke's Mid America Heart Institute, Kansas City, Missouri

^5^ Clinical Trial Centre Würzburg, University Hospital Würzburg, Germany;

^6^ Institute for medical Data Sciences, University Hospital Würzburg, Germany

**E-mail address of the corresponding author:** martha.nast@uni-wuerzburg.de

| **Table S1**: Results of the KCCQ-12 for both ways of administration (n = 61) | | | |
| --- | --- | --- | --- |
|  | **KCCQ (median, IQR)** | |  |
|  | **self - administered** | **telephone interview** | **p-Value** |
| **Domains** | | | |
| Physical Limitation^a^ | 83.33 (66.66 - 91.66) | 83.33 (58.33 – 100) | 0.96 |
| Symptom Frequency | 91.67 (66.66 – 95.83) | 83.33 (62.5 – 100) | 0.17 |
| Quality of Life | 75 (62.5 – 100) | 75 (62.5 – 87.5) | 0.54 |
| Social Limitations^a^ | 83.33 (50 – 100) | 83.33 (62.5 – 100) | 0.05 |
| **Scores** | | | |
| Clinical Summary Score | 85.42 (65.62 – 93.75) | 82.29 (67.70 – 93.75) | 0.40 |
| Overall Summary Score | 81.25 (65.62 – 93.75) | 83.33 (66.66 – 90.62) | 0.98 |
| KCCQ, Kansas City Cardiomyopathy Questionnaire; IQR, interquartile range, ^a^n = 60 | | | |

| **Table S2:** Test-retest reliability between telephone-assessed and self-administered KCCQ-12 (n = 61) | | |
| --- | --- | --- |
|  | **ICC** | **95%-CI** |
| **Domains** | | |
| Physical Limitation^a^ | 0.67 | 0.64 – 0.71 |
| Symptom Frequency | 0.77 | 0.74 – 0.80 |
| Quality of Life | 0.83 | 0.79 – 0.86 |
| Social Limitations^a^ | 0.71 | 0.67 – 0.74 |
| **Scores** | | |
| Clinical Summary Score | 0.83 | 0.79 – 0.86 |
| Overall Summary Score | 0.85 | 0.82 – 0.88 |
| KCCQ, Kansas City Cardiomyopathy Questionnaire; ICC, non parametric intraclass correlation coefficient; CI, confidence interval, ^a^n = 60. | | |


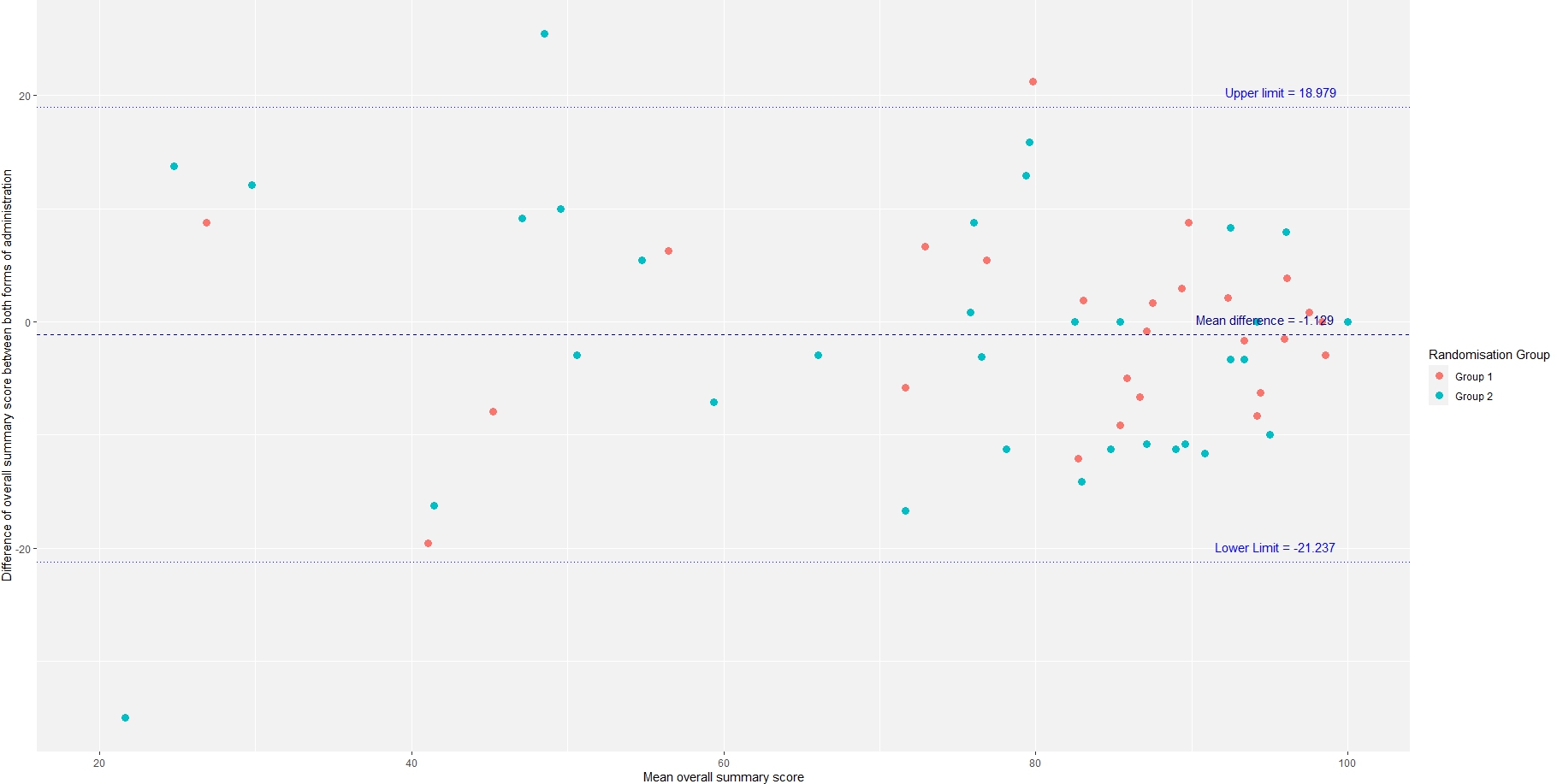


**Figure S1** Comparison of the median overall summary score with the difference of the overall summary score between both forms of administration, according to randomization group

Group 1: *self-administered questionnaire first; Group 2: telephone interview first.*


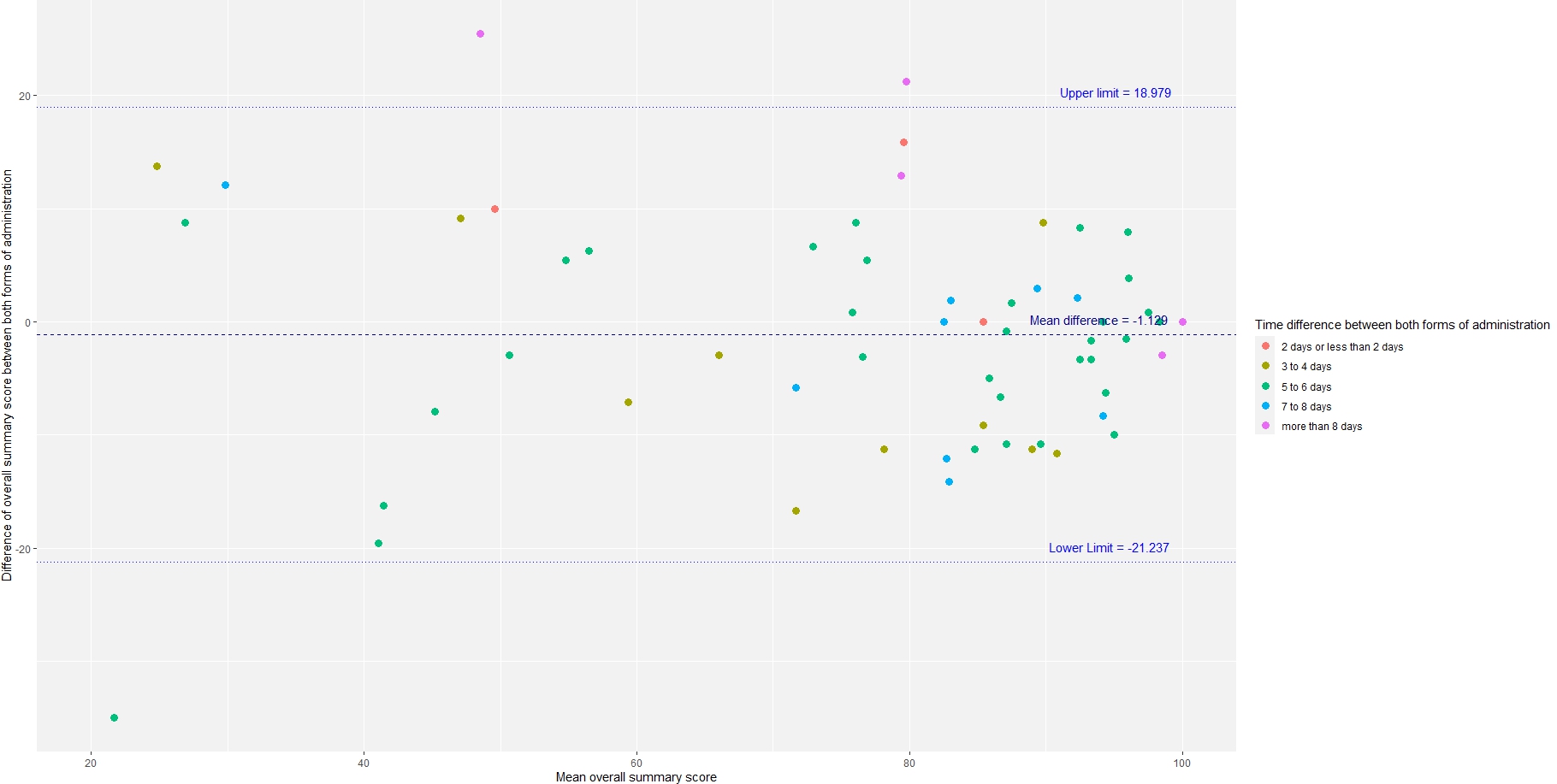


**Figure S2** Comparison of the median overall summary score with the difference of the overall summary score between both forms of administration, according to time interval between administrations
